# A Brief Account of the Hospital at Vienna, in a Letter to Professor Flint

**Published:** 1856-07

**Authors:** Edward L. Holmes

**Affiliations:** Buffalo, N. Y.


### ART. II.—*A Brief Account of the Hospital at Vienna, in a Letter to Prof. Flint.* By EDWARD L. HOLMES, M. D., Buffalo, N. Y.

SIR: It is with pleasure that I comply with your request to give you some account of the Hospital at Vienna, and the plan of instruction adopted by the eminent men of our profession connected with it.

You have yourself visited similar institutions at Paris and London, and can, perhaps, understand why those who pursue medical studies at Vienna, are usually better pleased with the advantages there offered, than elsewhere.

It is certainly a great convenience at Vienna that the student finds all the material he can wish for his investigations, brought within the limits of a single hospital. This hospital is situated a short distance without the "city" proper. Its grounds are very extensive, and entirely enclosed by long rows of buildings, like a wall. Other rows of buildings extend at right angles with each other within this enclosure, which is then divided into ten large square courts, the largest of which contains, perhaps, eight acres of land. The passage from one court to another is under broad archways. These courts are well laid out into fine gravel walks, which pass under rows of thrifty trees, in the shade of which are placed benches for the benefit of convalescent patients. On each side of these walks are green plots, bordered by hedges. Nearly all the courts are also provided with fountains, which are kept constantly playing. The buildings are of brick, covered with a yellowish colored plaster.

As I was informed, there are about 120 wards, accommodating 3600 patients. The wards are large, plain, tolerably well ventilated and amply provided with conveniences for bathing. From the fact that the various diseases are so well classified in this hospital, and that the visits to the different clinics do not conflict with each other, the student can, if he chooses, spend nearly the entire day in receiving the best instruction at the bedside. The visits are from an hour and a half to two hours in length. Early in the morning are the visits to the wards for general practice, followed in succession by those to the surgical and ophthalmic departments. In the afternoon one can give his attention to diseases of the skin, syphilis, obstetrics and auscultation and percussion. The material is ample in each of these departments.

When you reflect that at Paris all the visits to the numerous "services" at the different hospitals, situated so far from each other, are at the same hour, you can appreciate the advantage of the system adopted at Vienna.

The clinical instructors certainly devote the greatest attention to the interests of the student.

Prof. Skoda has a small "service" of twenty-four beds. Every patient is thoroughly examined, from time to time, and the attention of the student carefully directed to every important phenomenon. His lectures at the bedside are very scientific, and at the same time practical. In making his examinations for physical signs, the Prof. always employs the ivory pleximeter and hammer for percussion, and the common stethoscope for auscultation. He is, without doubt, a most accurate observer, and one in whose word the greatest confidence can be placed. In several particulars his views differ from those usually received by other eminent writers. He certainly has collected a large mass of facts to substantiate his theories. Few have had more opportunities than he to examine after death the effects of disease upon patients, whom he had during life for a long time under his most careful observation. It is said he is a perfect "skeptic" in therapeutics, and one would almost judge so, to follow his treatment at the hospital, which is exceedingly simple. His classes seem to look upon him with the greatest reverence.

Prof. Oppolzer, clinical lecturer upon general practice, is thought by most persons to be a more pleasing instructor. No one can fail to be highly interested and instructed in listening to him. He adopts a plan similar to that formerly followed by M. Valleix, at "La Pitiè," Paris, the student being called upon to examine the patient, give his diagnosis and treatment, when the professor expresses his own views upon the case. In his wards the students have also, to a certain extent, charge of a number of patients. It is their duty to prepare a full history of the cases, adding such remarks of their own as they may choose, and from day to day report the condition of the patient. In case of death the student often prepares an account of the appearance of the different organs as seen at the autopsy, which is read on the next day in the ward. On such occasions the Prof. is often inclined to examine the student with the utmost rigor in every thing pertaining to the diagnosis, treatment and pathology. As far as possible, all microscopical and chemical examinations of urine and discharges from the stomach and bowels are conducted in the ward.

While at Vienna I gave little attention to surgery, and cannot speak much from my own observation. There is one circumstance worthy of consideration, showing the encouragement offered by the Austrian government to those who are willing to devote themselves to science. From the various provinces of the empire are yearly selected twelve young men, who, upon examination, having proved themselves worthy, are sent to Vienna to perfect themselves in practical surgery. They receive a small sum from the government. In the amphitheatre, at the hospital, they perform nearly all the operations upon the patients, the professor simply standing near to advise, if necessary. Previous to performing any operation in the hospital, these young men have almost unlimited opportunity to dissect and operate upon the cadaver. At a trifling expense any foreigner can be furnished with sufficient material for the study of anatomy and practical surgery.

The opportunities for obstetrical practice are probably inferior to none in the world. In the clinic, open to students, fifteen to twenty births occur daily. The same plan of thorough instruction at the bedside is here given in diagnosis, as revealed by the "toucher," also in conducting labor. The courses in instrumental delivery are superior to anything we have of the kind in this country; since in place of the "phantom," the operations are performed upon the cadaver, in which are placed the bodies of dead children, to be turned or delivered by forceps After an individual has taken this course, he is allowed to conduct such cases of labor as require the use of forceps or the perforator, which may occur during the time he is permitted to have charge of the wards. All ordinary cases of labor are conducted by midwives, except when students wish to do it themselves. At this hospital women, as far as I have seen, are never bandaged after labor. I was also informed that this is not done in private practice. The mothers are removed in a very short time from the "labor room" to the other wards, where they usually remain nine days before leaving the hospital. I have several times seen women but a few hours after delivery, instead of being carried, suffered to walk from the room, of which I have just spoken. The children soon after birth, are carried to the hospital to priests to be baptized. They are clothed in a manner somewhat peculiar. The blanket, or quilt, instead of being sufficiently loose for the child to have free use of its limbs, is folded quite tightly around its body, confining the arms to its side and keeping its legs perfectly straight. In addition to this, the whole bundle is firmly wound round with tape, reminding one very forcibly of the pictures we sometimes see of Indian pappooses. The mother, on leaving the hospital, can either take her child home or leave it to be carried to the foundling hospital, where it is reared and finally put to a trade, or, perhaps, enters the army.

The clinic of Prof. Hebra, for diseases of the skin, is particularly worthy of notice. He is said to be the best practical teacher in his branch in Europe. His lectures are very popular among the students. Every male patient appears entirely naked before his class, and while the Professor is speaking upon the case, passes in turn before each listener for inspection. As the number of his patients is very large, he is able to exhibit repeatedly to his class, many cases of nearly every disease, and compare one with another, when there are difficulties of diagnosis.

There are equal facilities for studying venerial diseases under Prof. Sigmund.

I had for some time been much interested in diseases of the eye. The clinics, at Vienna, are by no means so large as at Paris. Those who visit them, however, find enough to interest, particularly in the courses for operations and the use of the ophthalmoscope, which are very thorough. These courses are given not only by those connected with the hospital, but also by Dr. Jaeger, and by Prof. Stellwag, at the military hospital. The Professors at Paris, are not in the habit of giving such courses.

In addition to being able to attend the daily exercises conducted by the Professors as I have described, the student is permitted to go round with the assistant physicians, who make their visits late in the afternoon.

Of course no one would neglect, while at Vienna, to attend, as much as possible, the post-mortem examinations. It is in the autopsy room of this hospital, that Prof. Rokitansky has made so many important investigations, the results of which he has embodied in his great work upon Pathology. The law permits the examination of all patients who die in this hospital. Moreover, when a case of sudden death occurs in the city from doubtful causes, the body is examined by the Professor. The average number of daily autopsies is only five, although the mortality in the hospital is much larger. A minute record is made of the appearances in each case, after which the morbid specimens are carried to the lecture-room to be demonstrated to the class by Prof. Rokitansky. The pathological museum, at Vienna, although not particularly large, has become, through the labors of Prof. Rokitansky, very valuable and select. The private courses given at this museum by the assistant of Prof. R., in which the preparations are carefully demonstrated, are very instructive. I might here mention a circumstance, which at first appeared to me very strange. In the wards, upon the death of a patient, whose case has offered anything of interest, a notice is at once placed over his bed, giving the hour of the autopsy. Whether the notice is read by the remaining patients with the same reflections as by the students in attendance, may be doubtful.

At Vienna, in addition to this large public institution for medical and surgical instruction, there are several other similar institutions worthy of notice. The hospital for children, the military hospital and the military medical college, are open to students under certain restrictions. In the last institution is one of the most extensive and perfect cabinets of anatomical preparations in wax, in the world.

There is also a homœopathic hospital in the city. I was able to visit it regularly for three weeks only. I would have been pleased to do so longer, but as no remarks were ever made concerning the medicines given, and as the visits were very short, I had no means of judging concerning their therapeutics. All I can say is, that from day to day I could see no marked change in the condition of the patients. It is true, many of them slowly recovered. I saw, however, two cases of typhoid fever (which is called typhus in Vienna) and one case of pneumonia, which terminated fatally. The hospital is much neater, kept in better order than the larger one, and is under the most excellent management of the Sisters of Charity. Only four students were visiting it during the time I was there, although I was told the number was larger when the lectures were given. The patients are never much troubled during the visits. It can certainly do no patient any good to be subjected to so many repeated examinations from the professors and students as the patients at the general hospital are obliged to undergo.

As a city to be visited merely for amusements, Vienna is by no means to be compared to Paris. But I have never seen a person who, after having spent considerable time there in study, did not regret to leave. The means of recreation are ample, and the social relations among students are pleasanter than those usually enjoyed by foreigners at Paris. The surrounding country is delightful, and in almost every direction there are numerous places much frequented by students in their excursions. The hospital, however, to the medical man, is the chief object of attraction.

I am confident no one could visit the wards of this celebrated hospital, without feeling a regret that in our own country we have no similar institution, where those who devote themselves to the profession are able to fit themselves thoroughly, by *actual practice*, for the important duties incumbent upon them.

It is true, we have medical schools in abundance, and able teachers; a faithful student can do much in every branch of his profession. But where in our own country is there an institution in which all the young men who pass through its course of instruction, can acquire practical knowledge in physical exploration, sufficient to enable him to depend, with a firm enlightened confidence, upon his own skill and judgment? Or what institution have we where the classes are furnished with such practical instruction in operative surgery, that after three years all are really competent to perform many operations that may be presented to them! The same remarks apply to our courses in obstetrics and pathological anatomy.

Physicians are expected never to make mistakes; and should one be so unfortunate as to do so, he is sure to be called to strict account. This is as it should be, but when so much is expected, certainly sufficient opportunities should be placed at the student's command for thorough preparation.

Our general and states' governments are appropriating large sums of money for geological and other scientific investigations. Why should they not be equally liberal for the advancement of medical science, which, to say the least, is of as much importance to the public good as any other?

It is earnestly to be hoped that means may be adopted to secure more thorough practical courses in medical and surgical science, by which young men may escape the unpleasant necessity of entering blindly, as it were, upon their duties, and of gaining their experience during the first few years of their practice at the expense of the unfortunate individuals who may fall under their care.

Most respectfully,

EDWARD L. HOLMES.

Prof. Austin Flint, M. D.

